# Perioperative and Functional Outcomes of Robot-assisted Ureteroenteric Reimplantation: A Multicenter Study of Seven Referral Institutions

**DOI:** 10.1016/j.euros.2021.11.005

**Published:** 2022-01-03

**Authors:** Albert Carrion, Ahmed Aly Hussein, Daniel Eun, Abolfazl Hosseini, Josep Maria Gaya, Ronney Abaza, Xavier Bonet, Umar Iqbal, Randall A. Lee, Ziho Lee, Matthew Lee, Carles Raventos, Oriol Moreno, Joan Palou, Alberto Breda, Fernando Lozano, Francesc Vigués, Enrique Trilla, Khurshid A. Guru

**Affiliations:** a Department of Urology, Hospital of Vall Hebron, Autonomous University of Barcelona, Barcelona, Spain; b Department of Urology, Roswell Park Comprehensive Cancer Center, Buffalo, NY, USA; c Department of Urology, Lewis Katz School of Medicine at Temple University, Philadelphia, PA, USA; d Department of Molecular Medicine and Surgery, Karolinska Institutet, Stockholm, Sweden; e Department of Pelvic Surgery, Karolinska University Hospital, Stockholm, Sweden; f Department of Urology, Fundació Puigvert, Autonomous University of Barcelona, Barcelona, Spain; g Department of Urology, Ohio Health Dublin Methodist Hospital, Columbus, OH, USA; h Department of Urology, Hospital Bellvitge, University of Barcelona, Barcelona, Spain; i Department of Urology, University of Washington, Seattle, WA, USA

**Keywords:** Robot-assisted ureteroenteric reimplantation, Ureteroenteric stricture, Radical cystectomy, Postoperative complications, Bladder cancer

## Abstract

**Background:**

Open revision of ureteroenteric strictures (UESs) is associated with considerable morbidity. There is a lack of data evaluating the feasibility of robotic revisions.

**Objective:**

To analyze the perioperative and functional outcomes of robot-assisted ureteroenteric reimplantation (RUER) for the management of UESs after radical cystectomy (RC).

**Design, setting, and participants:**

A retrospective multicenter study of 61 patients, who underwent 63 RUERs at seven high-volume institutions between 2009 and 2020 for benign UESs after RC, was conducted.

**Outcome measurements and statistical analysis:**

Data were reviewed for demographics, stricture characteristics, and perioperative outcomes. Variables associated with being stricture free after an RUER were evaluated using a multivariate Cox regression analysis.

**Results and limitations:**

Among 63 RUERs, 22 were right sided (35%), 34 left sided (54%), and seven bilateral (11%). Twenty-seven (44%) had prior abdominal/pelvic surgery and five (8%) radiotherapy (RT). Thirty-two patients had American Society of Anesthesiologists (ASA) scores I–II (52%) and 29 ASA III (48%). Forty-two (68%) RUERs were in ileal conduits, 18 (29%) in neobladders, and two (3%) in Indiana pouch. The median time to diagnosis of a UES from cystectomy was 5 (3–11) mo. Of the UESs, 28 (44%) failed an endourological attempt (balloon dilatation/endoureterotomy). The median RUER operative time was 195 (175–269) min. No intraoperative complications or conversions to open approach were reported. Twenty-three (37%) patients had postoperative complications (20 [32%] were minor and three [5%] major). The median length of hospital stay was 3 (1–6) d and readmissions were 5%. After a median follow-up of 19 (8–43) mo, 84% of cases were stricture free. Lack of prior RT was the only variable associated with better stricture-free survival after RUER (hazard ratio 6.8, 95% confidence interval 1.10–42.00, *p* = 0.037). The study limitations include its retrospective nature and the small number of patients.

**Conclusions:**

RUER is a feasible procedure for the management of UESs. Prospective and larger studies are warranted to prove the safety and efficacy of this technique.

**Patient summary:**

In this study, we investigate the feasibility of a novel minimally invasive technique for the management of ureteroenteric strictures. We conclude that robotic reimplantation is a feasible and effective procedure.

## Take Home Message

This is the first multicenter study in the literature that specifically investigates the feasibility of robot-assisted ureteroenteric reimplantation for the management of ureteroenteric strictures. This minimally invasive procedure is feasible and could be an alternative to open revisions.

## 1 Introduction

Radical cystectomy (RC) with pelvic lymph node dissection and ileal urinary diversion is the standard of care for the management of muscle-invasive bladder cancer and refractory non–muscle-invasive bladder cancer [1]. Benign ureteroenteric strictures (UESs) occur in 3–19% of patients according to large cystectomy series and represent the most common cause for reoperations after RC [2], [3], [4]. Owing to the low success rate of the endourological approach, open ureteroenteric reimplantation is the gold standard treatment for UESs [5]. However, open revisions are challenging surgeries with a non-negligible risk of complications [6]. Recently, utilization of minimally invasive approaches for ureteral reconstructive procedures has increased. Although only few single-center series of robot-assisted ureteroenteric reimplantation (RUER) were reported previously, outcomes are promising [7]. The potential benefits of this procedure could be those related to the minimally-invasive approach as well as the three-dimensional vision, tremor filtering, ability to suture with precision, and use of indocyanine green (ICG).

Herein, we aimed to report the perioperative and functional outcomes of RUER for the management of UESs after RC in a large series of patients from seven institutions.

## 2 Patients and methods

### 2.1 Study population

A retrospective multicenter study was conducted in 61 patients who underwent 63 RUERs between January 2009 and February 2020 for benign UESs after RC. Patients who had other ureteroenteric complications (stone obstruction, ureteric tumor, stomal obstruction, or external compression) were excluded. Data were collected from seven institutions, and institutional review board approval was obtained. Strictures were diagnosed by a computed tomography (CT) scan during follow-up or as a consequence of worsening of renal function, pain, or urinary infection. Renal scintigraphy, pouchogram, and/or pyelography was used to confirm the stricture in some cases or to exclude asymptomatic patients who had dilated pelvicalyceal systems with no deterioration of renal function or evidence of infection.

### 2.2 Surgical technique

**Fig. 1:**
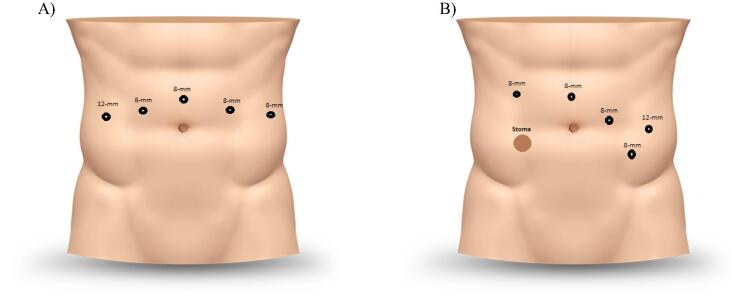
Trocar mapping: (A) neobladder diversion: an 8-mm supraumbilical port for camera, 8-mm ports in the right and left paraumbilical regions for the second and third arms, and 8-mm port in the left hypochondriac quadrant for the fourth arm; a 12-mm port in the right hypochondriac quadrant for the assistant if required. (B) Ileal conduit diversion: an 8-mm supraumbilical port for camera, an 8-mm port in the right hypochondriac quadrant for the second arm, an 8-mm port in the left paraumbilical region for the third arm, and an 8-mm port in the left iliac quadrant for the fourth arm; a 12-mm port in the left lumbar region for the assistant if required.

Supine or lithotomy in Trendelenburg position was utilized. Four 8-mm robotic ports were placed transperitoneally in triangulation around the internal end of the ileal conduit or in the same way as in robot-assisted radical cystectomy (RARC) in case of a neobladder (Fig. 1) [8]. One assistant port of 12 mm and another of 5 mm could also be used. Extensive adhesiolysis was often necessary to identify the key anatomical components. Intraureteral or intradiversion administration of ICG as described by Lee et al. [9] could help identify anatomical landmarks and the site of stricture. Ureterolysis was performed circumferentially toward the urinary diversion. The dilated ureter proximal to the stricture was dissected and transected. The distal extent of the UES was excised for pathology. The intravenous ICG injection could help in identifying healthy distal ureteral tissue [10]. The healthy end of the ureter was spatulated and reimplanted at a new site on the diversion using monofilament absorbable running or interrupted suture (4-0 or 5-0). For unilateral UESs, a Bricker anastomosis was recommended, and for bilateral UESs either a Wallace or a Bricker anastomosis could be used. After completing the posterior half of the anastomosis, a ureteral stent was placed transabdominally across the anastomosis, with a guidewire inserted through the assistant port. The proximal end of the stent was advanced till the renal pelvis, and the distal end was inserted into the urinary diversion under direct visualization. In ileal conduit cases, the stent could be placed retrogradely through a sucker placed in the conduit. A nephroureteral stent was used for ileal conduits, while both nephroureteral and double-J stents could be used for neobladders.

### 2.3 Study outcomes

Data were reviewed for demographics and perioperative outcomes. Previous abdominal/pelvic surgery (apart from RC) or radiotherapy (RT) was reported. We also reviewed patients who failed endourological management, defined as balloon dilatations or endoureterotomies. Perioperative parameters assessed were reimplantation side, stricture length (by antegrade pyelography), conversion to open approach, operative time (OT), use of ICG (intraureteral, intraurinary diversion, or intravenous), intra- and postoperative complications, length of hospital stay (LOS), and hospital readmission within the 1st month after RUER. Treatment failure (UES recurrence) was defined as radiological and/or clinical signs of recurrent obstruction requiring renal drainage, endourological or revision of reimplantation, or nephrectomy. All procedures were performed using the da Vinci robot.

### 2.4 Statistical analysis

Categorical variables were reported as frequencies and proportions, and continuous data as medians and interquartile range (IQR). Proportions, such as success rates and stricture characteristics, were compared by the Pearson chi-square test. Kaplan-Meier analysis and the log-rank test were used to compare the likelihood of stricture-free survival after RUER. Multivariate stepwise Cox regression analysis was fit to evaluate predictors of being stricture free after RUER. Statistical significance was considered at *p* = 0.05. Statistical analysis was done using SPSS version 20 (IBM Corp., Armonk, NY, USA).

## 3 Results

### 3.1 Clinicodemographic characteristics

A total of 63 RUERs were performed in 61 patients with a median age of 67 yr (IQR 60–72 yr); 22 strictures were right sided (35%), 34 were left sided (54%), and seven were bilateral (11%). Two patients underwent a separate revision for each side. Twenty-seven patients (44%) had a prior abdominal/pelvic surgery and five (8%) had prior abdominal/pelvic RT. Of the patients, 26 (43%) had an American Society of Anesthesiologists (ASA) score of II and 29 (48%) had ASA III. The RC approach was open in nine (15%), laparoscopic in two (3%), and robot-assisted in 50 (82%) patients. Among the RARC cases, ten (20%) underwent extracorporeal urinary diversion (ECUD) and 40 (80%) underwent intracorporeal urinary diversion (ICUD). Of the RUERs, 42 (68%) were in ileal conduits, 18 (29%) in ileal neobladders, and two (3%) in Indiana pouch (Table 1).

**Table 1:** Clinicopathological characteristics of 61 patients treated with RUER

Age (yr), median (IQR)	67 (60–72)
Gender, *n* (%)	
Male	50 (82)
Female	11 (18)
BMI, median (IQR)	28.3 (25.7–33.6)
ASA score, *n* (%)	
I	6 (9.8)
II	26 (42.6)
III	29 (47.5)
Creatinine (mg/dl), median (IQR)	1.32 (0.83–1.45)
eGFR (ml/min), median (IQR)	72.2 (47.5–88.3)
Prior abdominal/pelvic surgery other than RC, *n* (%)	27 (44.3)
Prior abdominal/pelvic radiotherapy, *n* (%)	5 (8.2)
Neoadjuvant chemotherapy, *n* (%)	25 (41)
Cystectomy approach, *n* (%)	
Open	9 (14.8)
Laparoscopic	2 (3.3)
Robot assisted	50 (82)
Type of urinary diversion, *n* (%)	
Ileal conduit	42 (68.8)
Neobladder	17 (27.8)
Indiana pouch	2 (3.2)
Urinary diversion approach a, *n* (%)	
Extracorporeal	10 (20)
Intracorporeal	40 (80)
Type of neobladder, *n* (%)	
Studer	11 (64.7)
Padovana	3 (17.6)
Hautmann	3 (17.6)
Overall death, *n* (%)	8 (13.1)
Follow-up, median (IQR)	36 (21–66.5)

ASA = American Society of Anesthesiologists physical classification; BMI = body mass index; eGFR = estimated glomerular filtration rate; IQR = interquartile range; RC = radical cystectomy; RUER = robot-assisted ureteroenteric reimplantation.

a Among robot-assisted cystectomy cases.

### 3.2 Perioperative characteristics and outcomes

The median time from cystectomy to stricture diagnosis was 5 mo (IQR 3–11 mo). Twenty-eight cases (44%) failed initial endoscopic and/or percutaneous management. The median time between the diagnosis and reimplantation was 4 mo (IQR 2–12 mo) and the median stricture length was 2 cm (IQR 1–3 cm). The pathological report of the resected ureter showed benign tissue in all cases. The median OT was 195 min (IQR 175–269 min). No intraoperative complications, conversions to open approach, or intraoperative blood transfusions were reported. Two patients received a blood transfusion after the surgery as a consequence of the intraoperative blood loss, but none of them had signs of postoperative active bleeding. Twenty-three patients (37%) had postoperative complications, of which 20 (32%) were minor (Clavien I–II) and three (5%) were major (Clavien III). The minor complications comprised urinary tract infections, ileus (managed conservatively), and urine leak (managed with prolonged stenting). The major complications included a femoral arteriovenous fistula (secondary to an anesthesia line placement), bilateral pulmonary embolisms requiring anticoagulation, and percutaneous nephroureteral stent placement after nephroureteral stent dislodgement. The median LOS was 3 d (IQR 1–6 d), and hospital readmissions occurred in 5% (Table 2).

**Table 2:** Perioperative characteristics and outcomes of 63 RUER procedures

Reimplantation side, *n* (%)	
Left	34 (54)
Right	22 (34.9)
Bilateral	7 (11.1)
Time to stricture formation a (mo), median (IQR)	5 (3–11)
Stricture length (cm), median (IQR)	2 (1–3)
Renal scintigraphy, *n* (%)	37 (58.7)
Anterograde pyelography, *n* (%)	57 (90.5)
Preoperative urinary drainage, *n* (%)	62 (98.4)
Nephrostomy tube	47 (75.8)
Ureteral catheter	15 (24.2)
Failed endoscopic procedure, *n* (%)	28 (44.4)
Patients with ICG use, *n* (%)	16 (25.4)
Intraureteral ICG	11 (17.4)
Intraurinary diversion ICG	1 (1.58)
Intravenous ICG	6 (9.5)
Operative time (min), median (IQR)	195 (175–269)
Conversion to open approach, *n* (%)	0 (0)
Intraoperative complications, *n* (%)	0 (0)
Intraoperative blood transfusion, *n* (%)	0 (0)
Postoperative complications, *n* (%)	23 (36.5)
Type of postoperative complication b, *n* (%)	
Postoperative blood transfusion	2 (3.2)
Urinary tract infection	8 (12.7)
Wound infection	1 (1.6)
Septic shock	0 (0)
Bleeding	0 (0)
Incisional hernia	7 (11.1)
Urine leak	7 (11.1)
Bowel injury	0 (0)
Ileus	5 (7.9)
Pulmonary embolism	1 (1.6)
Femoral arteriovenous fistula	1 (1.6)
Nephroureteral stent dislodgement	1 (1.6)
Length of ileus (d), median (IQR)	4 (2.5–9.5)
Clavien-Dindo, *n* (%)	
Minor (I–II)	20 (21.7)
Major (III)	3 (4.8)
Length of hospital stay (d), median (IQR)	3 (1–6)
Hospital readmission, *n* (%)	3 (4.8)
30-d creatinine (mg/dl), median (IQR)	1.36 (1–1.87)
30-d eGFR (ml/min), median (IQR)	49.5 (36.9–61.7)
Overall success, *n* (%)	53 (84.1)
Time to failure (mo), median (IQR)	13 (6.25–35)
Follow-up (mo), median (IQR)	19 (8–43)

eGFR = estimated glomerular filtration rate; ICG = indocyanine green; IQR = interquartile range; RUER = robot-assisted ureteroenteric reimplantation.

a Time between the cystectomy and the first diagnosis of stricture.

b Data are presented for each individual complication.

**Fig. 2:**
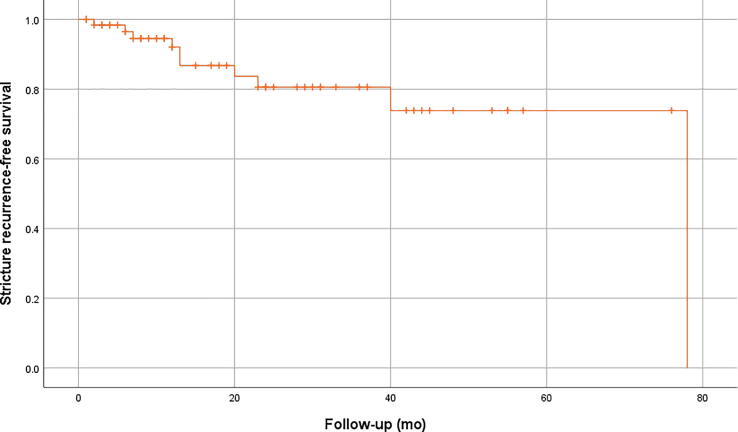
Kaplan-Meier analysis of ureteroenteric stricture-free survival after RUER of the overall cohort. RUER = robot-assisted ureteroenteric reimplantation.

After a median follow-up of 19 mo (IQR 8–43 mo), ten patients (16%) developed recurrent UESs with a median time between reimplantation and recurrence of 13 mo (IQR 6–35 mo). On Kaplan-Meier analysis, the estimated 3-yr stricture-free survival after RUER was 84% (Fig. 2).

### 3.3 Predictors of success

Table 3 shows the univariate analysis of the potential predictors of success. Patients with prior abdominal/pelvic RT had a success rate of 60% compared with 86.2% in those without RT (*p* = 0.12). A multivariate Cox regression analysis for assessing potential variables associated with stricture-free survival after RUER showed that the lack of prior abdominal/pelvic RT was the only significant variable (hazard ratio 6.80, 95% confidence interval 1.10–42.00, *p* = 0.037; Table 4).

**Table 3:** Univariate analysis for stricture-free rate in relation to perioperative characteristics

	% Stricture free (no. of procedures/total no.)	*p* value
Gender		0.255
Male	86.5 (45/52)	
Female	72.7 (8/11)	
BMI		0.92
≤30	83.8 (31/37)	
>30	84 (21/25)	
Prior abdominal/pelvic surgery		0.784
No	85.3 (29/34)	
Yes	82.8 (24/29)	
Prior abdominal/pelvic radiotherapy		0.124
No	86.2 (50/58)	
Yes	60 (3/5)	
Neoadjuvant chemotherapy		0.541
No	86.5 (32/37)	
Yes	80.8 (21/26)	
Type of urinary diversion		0.268
Ileal conduit	79.1 (34/43)	
Neobladder	94.4 (17/18)	
Indiana pouch	100 (2/2)	
Reimplantation side		0.506
Left	88.2 (30/34)	
Right	81.8 (18/22)	
Bilateral	71.4 (5/7)	
Preoperative urinary drainage		0.735
Nephrostomy tube	83 (39/47)	
Ureteral catheter	86.7 (13/15)	
Failed endoscopic procedure		0.28
No	88.6 (31/35)	
Yes	78.6 (22/28)	
ICG use (any route of administration)		0.913
No	84.4 (38/45)	
Yes	83.3 (15/18)	
Intravenous ICG use		0.955
No	84.2 (48/57)	
Yes	83.3 (5/6)	

BMI = body mass index.

**Table 4:** Multivariate Cox proportional regression analysis of stricture-free rate in relation to preoperative characteristics

	HR (CI 95%)	*p* value
Gender	2.5 (0.5–11.2)	0.214
Prior abdominal/pelvic surgery	1.9 (0.4–7.6)	0.357
Prior abdominal/pelvic radiotherapy	6.8 (1.1–42)	0.037 *
Reimplantation side	1.5 (0.4–4.9)	0.468
Failed endoscopic procedure	1.3 (0.3–5.1)	0.678

CI = confidence interval; HR = hazard ratio.

* *p* < 0.05.

## 4 Discussion

To our knowledge, this is the largest series of RUER for UESs after RC. A benign UES is a common complication that can lead to a variety of health conditions and impairment of quality of life. Management of this complication is challenging, with several options described in the literature but without specific recommendations in clinical guidelines. UESs can occur in up to 19% of patients after RC. Previous studies reporting UESs varied in the surgical technique or experience, and the frequency and duration of follow-up [11], [12]. Prior abdominal RT is a well-known risk factor for UESs [13]. Our series included both open RC (ORC) and RARC patients. Prospective randomized controlled trials have shown similar perioperative outcomes between ORC and RARC, while a recent prospective study demonstrated no difference in stricture rate [14], [15]. However, the association between RARC (ICUD or ECUD) and UESs is still unclear. It was suggested that ECUD could increase the risk of UESs as a consequence of the higher dissection of the ureters compared with ICUD [16]. In contrast, Ahmed et al. [12] found a higher rate of UESs after ICUD, especially during their early learning curve.

It has been demonstrated that the endoscopic procedures have poor long-term efficacy for the management of UES [17]. Nevertheless, it is usually used as the initial management in order to avoid the morbidity of an open surgery and the difficulty associated with operating in a previously operated abdomen. Gin et al. [18] suggested that prior endoscopic attempts could worsen the outcomes during subsequent ureteroenteric reimplantation and recommended performing a primary open/robotic revision. Although prior failed endoscopic attempts were not a predictor of recurrence in our study, >50% of our patients underwent a primary RUER. Our findings suggest that, currently, the primary robotic revision of a UES could be increasing as a result of its low morbidity in the hands of experienced surgeons.

As a consequence of the low efficacy of conservative procedures, open revision is considered the gold standard treatment for UESs [17]. The success rate of open surgical revision is up to 85% [5], [18], [19], although it could decrease to 78% at a longer-term follow-up [20]. The intra-abdominal adhesions with the risk of bowel injury and the surrounding major vessels around the stricture area could deter surgeons from performing this procedure. With the development of minimally invasive techniques, multiple small single-center series of RUER have shown a success rate of up to 80% [7], [9], [12], [21]. A unique case of successful RUER performed by using a pure single-site approach (daVinci SP) and the initial experience of 11 laparoscopic ureteroenteric reimplantations have been also reported [22], [23]. However, the role of robotic surgery in the management of UESs is still undefined, and there is a lack of studies comparing the outcomes between open and robotic revisions. We found that 84% were stricture free 19 mo after RUER, demonstrating that this approach could be a reasonable alternative to open surgery. Our work is the first multicenter study in the literature that specifically investigates the feasibility of this procedure in a relatively large series of patients.

Several studies have reported a high rate of postoperative complications following open management of UESs. A summary of the most important series reporting on open revisions of UESs is shown in Supplementary Table 1. Packiam et al. [6] found considerable perioperative morbidity in a large series of open revision patients, reporting 48% of postoperative complications, of which 12% were major [6]. Two studies compared postoperative complications between open and robotic revisions. One reported 33% of postoperative complications among 45 patients who underwent an open revision compared with zero in five robotic procedures [18]. The other showed comparable perioperative outcomes between six open and 16 robotic approaches [12]. A small number of robotic procedures were included in both studies. In contrast, a recent series of eight patients treated with RUER reported five cases with postoperative complications. Despite the small sample, the authors concluded that ureteroenteric reimplantation might be a morbid procedure regardless of the operative approach [9]. In our series, postoperative complications occurred in 37% and only three (5%) were major. These results support the belief that although RUER is a morbid surgery, the risk of high-grade complications is lower than in the open approach. RUER has also been associated with a shorter LOS than open reimplantation (3 vs 6–10 d) [5], [16], [18]. In the current study, the median LOS was 3 d with a readmission rate of 5%. However, the low number of patients, and the variety in population and surgeons might decrease the generalizability and strength of our findings.

Identification of the ureters and urinary diversion in UES patients could be challenging because of the intraperitoneal adhesions and periureteral fibrosis. The use of ICG (nontoxic tracer) could facilitate the identification of the ureter and the site of the stricture. Two studies reported the outcomes of eight and ten patients, who underwent RUER with the use of ICG injected in the ureter. They suggested that this approach provided a quick and precise identification of the ureter and could reduce the risk of vascular and bowel injuries [7], [9]. Although ICG was injected into the ureter only in 17% of our procedures, we did not report any case of conversion to open approach or intraoperative complication in the overall cohort. On the contrary, a UES is most commonly attributed to compromised vascularity of the ureter. A recent study reported a significant reduction in the UES rate following the implementation of intravenous (IV) ICG during RARC [10]. Even though we agree that this maneuver is useful for the assessment of distal ureter vascularization, we did not find a higher success rate in patients who received intravenous ICG. However, our results have several limitations, including the low number of patients who received IV ICG (10%) and that they were all from the same institution. ICG is helpful in assessing the vascularity only when injected IV rather than intraureteral. Prior intraureteral ICG may preclude its benefit to assess vascularity.

Limitations of robotic surgery remain the difficulty of access in a previously operated abdomen and the lack of tactile feedback. Dangle and Abaza [21] suggested that patients who had undergone previous RARC rather than ORC may had reduced intraperitoneal adhesions, and this in fact could facilitate ureteroenteric reimplantation. Our series included mainly patients having undergone minimally invasive RC, and the reported results are hardly comparable with other results available in the literature. Nevertheless, although nine and ten patients of our series were previously treated with ORC and RARC with ECUD, respectively, none of them had to be converted to an open approach or had intraoperative complications. We believe that RUER is feasible with either approach to RC.

In accordance with previous studies of open revisions, we found that the success rate of RUER did not differ by reimplantation side, failed endoscopic attempt, type of urinary diversion, and previous abdominal surgery [5]. One study reported worse outcomes in open revisions among patients who had a preoperative nephroureteral catheter than in those with percutaneous nephrostomy, in contrast to our findings [6]. We used both univariate analysis and multivariate Cox regression analysis due to the low event rate. On multivariate analysis, lack of prior abdominal RT was the only variable significantly associated with being stricture free after RUER. Given the difficulty of operating in a previously irradiated abdomen and the lower success rate, patients should be counseled about the risk-benefits of undergoing RUER. It has been suggested that successful endourological treatment for stricture depends primarily on stricture length [5]. This finding was not confirmed in prior open or robotic series, and was not evaluated in the current study as it was missing for many patients.

To our knowledge, this is the largest report of RUER. However, several limitations exist. The retrospective nature has its known limitations. Another limitation is the variation among institutions in surgical volume, experience, surgical technique, and follow-up protocols. However, all the procedures were performed at high-volume centers using high-volume robotic surgeons who followed similar standardized surgical techniques. Furthermore, our study is strengthened by the fact that it is difficult to generate a large single-institutional series of RUER with optimal follow-up. The relatively small number of patients and the variety in population can limit generalizability of the results.

## 5 Conclusions

RUER is a feasible procedure for the management of UESs. Prospective and larger studies are warranted to prove the safety and efficacy of this technique.

  ***Author contributions*:** Enrique Trilla had full access to all the data in the study and takes responsibility for the integrity of the data and the accuracy of the data analysis.

*Study concept and design*: Carrion, Eun, Raventos, Guru.

*Acquisition of data*: Carrion, Hussein, Hosseini, Gaya, Abaza, Bonet, Iqbal, Z. Lee, M. Lee, Moreno.

*Analysis and interpretation of data*: Carrion, Lozano.

*Drafting of the manuscript*: Carrion, Hussein, Eun, Lozano, Trilla.

*Critical revision of the manuscript for important intellectual content*: Carrion, Hussein, Hosseini, Abaza, Palou, Breda, Vigués, Guru.

*Statistical analysis*: Carrion, Hussein, Raventos.

*Obtaining funding*: None.

*Administrative, technical, or material support*: Carrion, Iqbal, R.A. Lee.

*Supervision*: Carrion, Eun, Trilla, Guru.

*Other*: None.

  ***Financial disclosures:*** Enrique Trilla certifies that all conflicts of interest, including specific financial interests and relationships and affiliations relevant to the subject matter or materials discussed in the manuscript (eg, employment/affiliation, grants or funding, consultancies, honoraria, stock ownership or options, expert testimony, royalties, or patents filed, received, or pending), are the following: None.

  ***Funding/Support and role of the sponsor*:** None.
