# Supplementary material for: Perioperative and Functional Outcomes of Robot-assisted Ureteroenteric Reimplantation: A Multicenter Study of Seven Referral Institutions
Source: Eur Urol Open Sci. 2022 Jan 3;35:47–53. doi: 10.1016/j.euros.2021.11.005 (PMC8738891; doi:10.1016/j.euros.2021.11.005)
Supplement: Supplementary data 1 [file mmc1.docx]

| Supplementary Table 1. Summary of the most important series reporting on open revisions of UES. | | | | | | |  |  | |  |  | |  |  |
| --- | --- | --- | --- | --- | --- | --- | --- | --- | --- | --- | --- | --- | --- | --- |
| Study | No. of patients | | Ileal counduit/Neobladder/Other (%) | | Intraoperative complications (%) | | | Overall postoperative complications / major (%) | | LOS* (days) | Success rate (%) | |  |  |
| Packiam et al.^6^ | 124 | | 31/56/13 | | 14 | | | 48/12 | | 6 | 93 | |  |  |
| Schöndorf et al.^5^ | 35 | | 26/63/11 | | NR | | | 18/NR | | 10 | 91 | |  |  |
| Nassar et al. ^20^ | 32 | | 22/66/12 | | NR | | | NR | | 8 | 84 | |  |  |
| Laven et al. ^17^ | 15 | | 7/86/14 | | 13 | | | 20/6 | | 6 | 80 | |  |  |
| Msezane et al. ^19^ | 41 | | 15/75/10 | | NR | | | NR | | NR | 88 | |  |  |
| Gin et al. ^18^ | 37 | | 35/43/22 | | NR | | | 38/3 | | 6 | 100 | |  |  |
| *LOS= length of hospital stay | |  | |  | |  | | |  | | |  | |  |
